# First Report of *Clostridium difficile* NAP1/027 in a Mexican Hospital

**DOI:** 10.1371/journal.pone.0122627

**Published:** 2015-04-27

**Authors:** Adrián Camacho-Ortiz, Daniel López-Barrera, Raúl Hernández-García, Alejandra M. Galván-De los Santos, Samantha M. Flores-Treviño, Jorge M. Llaca-Díaz, Héctor J. Maldonado Garza, Francisco J. Bosques-Padilla, Elvira Garza-González

**Affiliations:** 1 Servicio de Infectología, Hospital Universitario Dr. José Eleuterio González, Universidad Autónoma de Nuevo León, Monterrey, Nuevo León, Mexico; 2 Servicio de Gastroenterología, Hospital Universitario Dr. José Eleuterio González, Universidad Autónoma de Nuevo León, Monterrey, Nuevo León, Mexico; 3 Departamento de Patología Clínica, Hospital Universitario Dr. José Eleuterio González, Universidad Autónoma de Nuevo León, Monterrey, Nuevo León, Mexico; Charité, Campus Benjamin Franklin, GERMANY

## Abstract

**Background and Objective:**

*Clostridium difficile* NAP1/ribotype 027 is associated with severe disease and high mortality rates. Our aim was to determine the prevalence of NAP1/ribotype 027 among *C*. *difficile* isolates in a tertiary care hospital, and review the main clinical data.

**Methods:**

We included 106 stool samples from 106 patients. Samples were tested for A&B toxins and were cultured on CCFA agar. The genes *tcdA*, *tcdB*, *tcdC*, *cdtA*, and *cdtB* were amplified using PCR in clinical isolates. The *tcdA* 3’-end deletion analysis, PCR-ribotyping, and pulsed-field gel electrophoresis (PFGE) were also performed. Stool samples that were positive for culture were tested by the GeneXpert *C*. *difficile* assay. Clinical data were collected.

**Results:**

Thirty-six patients tested positive for A&B toxins; and 22 patients had positive culture for *C*. *difficile*, 14 of which tested positive for the A&B toxins and all 22 patients tested positive by the GeneXpert *C*. *difficile* assay. Risk factors included an average hospital stay of 16.1 days prior to toxin detection, average antibiotic use for 16.2 days, and a median of 3 antibiotics used. The 30-day crude mortality rate was 8.4%. Six of the 22 patients died, and 3 of those deaths were directly attributed to *C*. *difficile* infection. The majority of isolates, 90.9% (20/22), carried genes *tcdB*, *tcdA*, *cdtA*, and *cdtB*; and these strains carried the corresponding downregulator gene *tcdC*, with an 18-bp deletion. PFGE was performed on 17 isolates, and one main pattern was observed. Analysis of the ribotyping data showed similar results.

**Conclusion:**

The above findings represent the clonal spread of *C*. *difficile* in our institution, which mainly includes the NAP1/027 strain. This is the first report of *C*. *difficile* ribotype NAP1/027 in Mexico.

## Introduction

*Clostridium difficile* is a Gram-positive anaerobic bacterium that is able to produce spores. This bacterial species causes a potentially fatal diarrheal disease due to the production of its principal virulence factors toxin A and toxin B, which are encoded by their genes *tcdA* and *tcdB* that are located on a 21-kilobase section of chromosomal DNA known as the pathogenicity locus (*PaLoc*). The *tcdC* gene is thought to encode a negative regulator of toxin production. Therefore, enhanced toxin production, and thus increased virulence, is often derived in strains with an aberrant *tcdC* genotype. In addition to toxins A and B, some strains also produce a third toxin known as binary toxin, encoded by *ctdA* and *ctdB*, located outside the *PaLoc* [1]. The spores of this anaerobic bacterium are widely distributed in hospital environments, and their ingestion by patients with an altered gut microflora contributes to colonization and disease [1,2].

The spectrum of *C*. *difficile* infection (CDI) is very wide, starting from asymptomatic carriage to severe diarrhea that may progress to pseudomembranous colitis and toxic megacolon. The epidemiology of *C*. *difficile* has changed in the past decade, and a new type has emerged: polymerase chain reaction (PCR) ribotype (RT) 027/North American Pulsed (NAP)-field type 01. Major outbreaks associated with this strain have been described since 2004, first in Canada followed by the USA and Europe [1,3–5]. *C*. *difficile* NAP1/027 is associated with a severe disease presentation and high morbidity and mortality rates; therefore, it presents a major clinical and financial burden [6]. In Latin America, this strain has been found in Costa Rica and more recently in Chile [7]. In Mexico, studies have shown the clinical characteristics of patients with CDI demonstrating a significant risks of developing CDI following the use of H2 blockers, if they had a prior hospitalization within 12 weeks of diagnosis, if they had been in the intensive care unit, prior use of cephalosporin’s, fluoroquinolones [7] and clindamycin [7] but genetic analysis of Mexican isolates have not been published. Thus, our aim was to determine the prevalence of NAP1/027 among *C*. *difficile* isolates in a tertiary care hospital, and review the main clinical data.

## Material and Methods

### Ethics Statement

This study was performed with the approval of the Local Ethics Committee of the School of Medicine of the Universidad Autónoma de Nuevo León (Approval MB11-007). Written informed consent approved by the Ethics Committee was obtained from all patients.

When applied, written informed consent was obtained from caretakers, or guardians on behalf of the minors enrolled in this study.

### Study population and collection of data

From March 2011 through August 2012, 106 stool samples were analyzed from hospitalized patients aged 16 years or older. We enrolled patients with a hospital stay greater than 48 h or a recent hospitalization (in the previous 12 weeks) who developed diarrhea (3 or more depositions in the last 24h with a Bristol score of 6 or 7) and in whom there was no obvious explanation of the cause. All subjects agreed to participate in the study by signing an informed consent.

The demographic and clinical data collected in the study were age, gender, and length of hospital stay. In addition, the usage and dosage of antibiotics within the past 2 months were investigated as potential risk factors for CDI. Laboratory tests such as white blood cell (WBC) count, creatinine, and albumin were performed within 24 h of enrollment.

### Detection of infection

The stool samples collected were tested for toxins using the ImmunoCard toxins A&B assay (Meridian Bioscience, Cincinnati, OH, USA). Furthermore, fecal samples were collected from patients and were treated with ethanol shock for 3 h prior to inoculation on cycloserine-cefoxitin fructose agar (CCFA). Incubation was achieved in an anaerobic atmosphere using a GasPak EZ anaerobe pouch system (Becton Dickinson, Sparks, MA, USA) at 37°C for 48 h; and anaerobic colonies were identified using catalase, Gram staining, and the Crystal Identification System (Becton Dickinson).

### Analysis of clinical isolates

For all isolates, multiple PCR were performed to genotype *tcdA*, *tcdB*, *cdtA*, *cdtB*, *tcd*C. We also detected the deletion in the downregulator gene *tcdC*.

To obtain DNA form clinical isolates, five colonies were transferred into 200 μL of 100 mM Tris-HCl, 150 μg of lysozyme was added, and the mixture was incubated at 37°C overnight. Finally, genomic DNA was extracted with a standard phenol-chloroform-isoamyl alcohol protocol.

### *tcdA*, *tcdB*, *cdtA*, and *cdtB* analysis

The genes *tcdA*, *tcdB*, *cdtA*, and *cdtB* were amplified using a multiplex PCR method [8]. The total volume was 25 μL, consisting of 2.5 μL of 10× PCR buffer, 100 ng of DNA, 3mM MgCl_2_, 200 μM of each dNTP, and 1 U of Taq polymerase (Bioline, London, UK), and the primers tcdA-F3345, tcdA-R3969, tcdB-F5670, tcdB-R6079A, tcdB-R6079B, cdtA-F739A, and cdtA-F739B were used (Table 1). Thermocycler conditions were as follows: 3 min at 94°C; then 35 cycles of 50 s at 94°C, 40 s at 56°C, and 50 s at 72°C; and a final extension of 3 min at 72°C.

**Table 1:** Primers used for typing *of C*. *difficile*.

	Forward and reverse primers (5'3')	Concentration (μM)
tcdA-F3345	GCATGATAAGGCAACTTCAGTGGTA	0.6
tcdA-R3969	AGTTCCTCCTGCTCCATCAAATG	0.6
tcdB-F5670	CCAAARTGGAGTGTTACAAACAGGTG	0.4
tcdB-R6079A	GCATTTCTCCATTCTCAGCAAAGTA	0.2
tcdB-R6079B	GCATTTCTCCGTTTTCAGCAAAGTA	0.2
cdtA-F739A	GGGAAGCACTATATTAAAGCAGAAGC	0.05
cdtA-F739B	GGGAAACATTATATTAAAGCAGAAGC	0.05
cdtA-R958	CTGGGTTAGGATTATTTACTGGACCA	0.1
cdtB-F617	TTGACCCAAAGTTGATGTCTGATTG	0.1
cdtB-R878	CGGATCTCTTGCTTCAGTCTTTATAG	0.1
C1	TTAATTAATTTTCTCTACAGCTATCC	0.3
C2	TCTAATAAAAGGGAGATTGTATTATG	0.3
tcdC-F252	CATGGTTCAAAATGAAAGACGAC	0.3
tcdC-R415	GGTCATAAGTAATACCAGTATCATATCCTTTC	0.3
NK9	CCACCAGCTGCAGCCATA	0.17
NKV011	TTTTGATCCTATAGAATCTAACTTAGTAAC	0.17
16S	GTGCGGCTGGATCACCTCCT	0.2
23S	CCCTGCACCCTTAATAACTTGACC	0.2

### *tcd*C analysis

PCR was performed to amplify the *tcd*C gene according to the method used by Spigaglia et al.(2002) [9]. The total volume was 25 μL, consisting of 2.5 μL of 10× PCR buffer, 100 ng of DNA, 3mM MgCl_2_, 200 μM of each dNTP, and 1 U of Taq polymerase. The primers used were C1 and C2 (Table 1). Thermocycler conditions were as follows: 5 min at 94°C; then 30 cycles of 1 min at 94°C, 1 min at 50°C, and 1 min at 72°C; and a final extension of 3 min at 72°C.

### *tcd*C internal in-frame deletion analysis

For *tcd*C internal in-frame deletion analysis, PCR was performed as described by Persson et al.(2011) [8]. The total volume was 25 μL, consisting of 2.5 μL of 10× PCR buffer, 100 ng of DNA, 3mM MgCl_2_, 200 μM of each dNTP, and 1 U of Taq polymerase. The primers used were tcdC-F252 and tcdC-R415 (Table 1). Thermocycler conditions were as follows: 3 min at 94°C; then 35 cycles of 50 s at 94°C, 40 s at 56°C, and 50 s at 72°C; and a final extension of 3 min at 72°C.

### *tcdA* 3’-end deletion analysis

For *tcdA*3’-end deletion analysis, the reaction mixture consisted of 25 μL, containing 2.5 μL of 10× PCR buffer, 100 ng of DNA, 3mM MgCl_2_, 200 μM of each dNTP, 1 U of Taq polymerase, and the primers NK9 and MNKV011 [10]. Thermocycler conditions were as follows: 6 min at 94°C; then 37 cycles of 20 s at 94°C, 30 s at 55°C, and 2 min at 60°C; and a final extension of 3 min at 60°C.

### GeneXpert *C*. *difficile*

The Xpert *C*. *difficile*/Epi assay (Cepheid, CA) was performed as described by the manufacturer. Briefly, each stool sample positive for culture was transferred into a sample reagent vial. The vial was vortexed for 10 s, and the solution was pipetted into the cartridge by using a Pasteur pipette. The cartridge was placed on the Xpert instrument, and the assay was performed using the GeneXpert *C*. *difficile* assay program.

### PCR-Ribotyping

Amplification reactions were performed according to the method described by Bidet et al.[11]. The reaction mixture consisted of 25 μL, containing of 2.5 μL of 10× PCR buffer, 100 ng of DNA, 3mM MgCl_2_, 200 μM of each dNTP, 1 U of Taq polymerase, and the primers 16S and 23S (Table 1). Thermocycler conditions were as follows: 6 min at 94°C; then 35 cycles of 1 min at 94°C, 1 min at 57°C, and 1 min at 72°C; and a final extension of 7 min at 72°C. Amplification products were subjected to electrophoresis through a 2.5% agarose gel stained with ethidium bromide for 6 h at 85 V and then analyzed on an ultraviolet table with the LabWorks Image Acquisition and Analysis Software Version 4.5.00.0 for Windows (UVP, Inc., Upland, CA, USA). *C*. *difficile* ATCC 9689 and ATCC 9689 were used as a control.

### Pulsed-Field Gel Electrophoresis (PFGE)

PFGE was performed according to the method used by Alonso et al. (2002), with minor modifications [12]. A bacterial culture was grown in CCFA agar three times before starting, and then it was inoculated into fluid thioglycollate medium (FTM) and grown for 24 h at 37°C. Next, 2 mL of the FTM mixture was taken and centrifuged to obtain the bacterial pellet. The pellet was washed one time with 1 mL of cell suspension buffer and then resuspended in 410 μL of cell suspension buffer. Each bacterial suspension was mixed with 150 μL of 1.5% agarose and mixed thoroughly. The mixture was transferred to plug molds (35 μL for each plug) and allowed to solidify at 4°C for 10–15 min. The solidified plugs were incubated in 1 mg mL^-1^ lysozyme solution (100 μL of 25 mg mL^-1^ lysozyme stock plus 2.5 mL of lysozyme buffer) for 2 h at 37°C. The lysozyme was removed, and the plugs were rinsed with sterile water. The plugs were incubated with 20 U mL^-1^ proteinase K solution (100 μL of >600 U mL^-1^ proteinase K stock in 2.5 mL of proteinase K reaction buffer) at 50°C overnight (17 h). Then, the plugs were washed four times with 1× washing buffer for 30 min at room temperature with agitation. Next, the plugs were incubated for 1 h with 1 mL of 1× restriction enzyme buffer at room temperature and then overnight in 300 μL of 1× restriction enzyme buffer containing 30–50 U of the restriction enzyme SmaI at 30°C. After digestion, the buffer with the restriction enzyme was removed and the plugs were incubated for 30 min in 1× washing buffer. The plugs were loaded on a 1% agarose gel with 0.5× Tris-borate-EDTA buffer with thiourea (200 μM). Electrophoresis was run at 6 V for 22 h with pulse times starting at 1 s and ending at 35 s using the CHEF-DR III Pulsed-Field Electrophoresis System (Bio-Rad, Hercules, CA, USA).

### Control strain

Toxigenic strain *Clostridium difficile* ATCC BAA-1805 was used as control strain in all assays.

### Statistical analysis

Descriptive analysis was performed. We used Wilcoxon sum test when comparing means and Fisher exact test or Chi square test for comparison of dichotomical variables. S.P.S.S ver 18 was used

## Results

### Clinical data of patients

**Fig 1:**
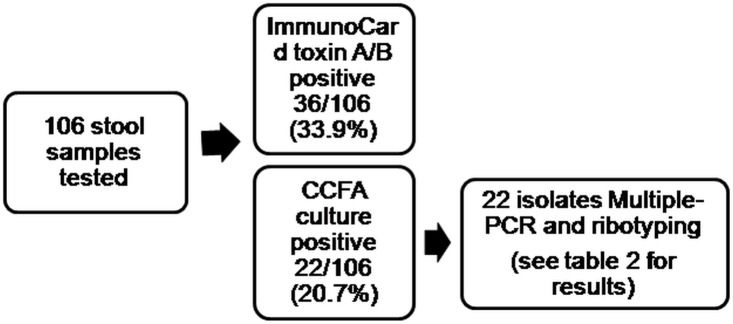
Distribution of tests performed in the study population.

During the 18-month surveillance period, we screened 106 patients with probable CDI, and 36 tested positive with the ImmunoCard assay. Furthermore, we found 22 patients with a positive culture for *C*. *difficile*, 14 of which tested positive with the Immuno Card assay and all 22 were positive by the GeneXpert assay. (Fig 1)

Of the 22 patients with a positive culture, 9 were women (40.9%) and the median age was 46.5 years old. Risk factors included an average hospital stay of 16.1 days prior to toxin detection, average antibiotic use for 13.5 days, and a median of 3 antibiotics used. The most common antibiotics used prior to toxin detection were third-generation cephalosporins (11/22), clindamycin (11/22), and flouroquinolones (7/22). Patients had a median of 9 stool movements per day. The average levels of peripheral leukocytes, albumin, and creatinine were 22,200 cells/mL, 2.0 g/dL, and 1.69 mg/dL, respectively. We observed differences in prior use of clindamycin and overall mortality between culture positive and culture negative patients (Table 2)

**Table 2:** Comparison of clinical characteristics between culture positive and culture negative patients.

Characteristic	Culture positive CDI (n = 22)	Culture negative (n = 84)	p
Male gender	14 (63.6)	55 (74.1)	0.978
Age	46.5 (15–89)	45.6 (15–79)	**0.035**
Median bowel movements on the day prior to diagnosis of CDI	9 (4–20)	4 (3–10)	0.189
Average BMI	23.0 (15.6–27.8)	25.9 (15.5–38.7)	0.997
Albumin (g/dL)	2.0 (1–3.2)	2.19 (0.9–3.7)	0.370
Creatinine (mg/dL)	1.69 (0.3–10.56)	0.95 (0.2–5.9)	0.408
Length of hospital stay	30.8 (7–115)	30.7 (1–77)	0.942
Days of antibiotic use before symptoms (median)	13.5 (0–49)	13.0 (0–90)	0.174
Third gen. cephalosporins	11 (50.0)	28 (32.5)	0.203
Clindamycin	11 (50.0)	19 (22.0)	**0.019**
Fluorquinolones	7 (31.8)	28 (32.5)	0.947
Prior ICU stay	1 (4.5)	13 (15.1)	0.336
Previous intrabdominal surgery	11 (50)	41(48.8)	0.920
Overall 30 day-mortality	6 (27.7)	3 (3.5)	**0.002**

CDI: *Clostridium difficile* infection; ICU: Intensive care unit. Values are given as n (%) or mean ± SD.

Treatment was combined (enteral vancomycin and IV metronidazole) in 9/22 (40.9%) patients, and 13/22 (59%) had monotherapy (10 metronidazole, 2 vancomycin, and 1 rifaximin). The overall 30-day crude mortality rate was 8.4% (9/106). Two patients required colectomies for treatment of CDI. Six of the 22 patients died, and 3 of those deaths were directly attributed to CDI.

### Isolates

Twenty-two isolates were collected from 22 patients (mean age, 51.7 years old; range, 20–89 years old; 10 females, 12 males). Most of these isolates were collected from medical wards, but other wards such as trauma and intensive care units were also involved (Table 3). Toxin gene profiles were assessed based on PCR, and two profile types were noted. The majority of isolates, 90.9% (20/22), carried genes encoding enterotoxin, cytotoxin, and the binary toxins (*tcdB*, *tcdA*, *cdtA*, and *cdtB*); and these strains carried the corresponding downregulator gene *tcdC*, with an 18-bp deletion. The 20 stool samples from which these isolates were obtained tested positive for the toxigenic NAP1/027 strain: toxigenic *C*. *difficile* positive/presumptive NAP1/027 positive.

**Table 3:** Presence and composition of the cytolethal distending toxin (CDT) locus in the *C*. *difficile* strains tested.

Isolate	*tcdA*	*tcdB*	*tcdC*	*tcdC* deletion	*cdtA*	*cdtB*	ImmunoCard toxins A&B	Ward	Collection date (m/d/y)	Age (y)	Gender
100	+	+	+	18	+	+	+	IM	11/9/11	84	Female
102	+	+	+	18	+	+	-	IM	11/14/11	64	Female
104	+	+	+	18	+	+	-	ICU	11/15/11	40	Male
106	+	+	+	0	-	-	+	ICU	12/22/11	59	Male
108	+	+	+	18	+	+	+	Surgery	11/28/11	64	Male
111	+	+	+	18	+	+	+	ER	12/2/11	52	Male
118	+	+	+	18	+	+	-	GS	1/6/12	57	Male
121	+	+	+	18	+	+	+	Trauma	1/31/12	46	Male
122	+	+	+	18	+	+	+	IM	2/8/12	21	Female
127	+	+	+	18	+	+	-	ICU	3/2/12	86	Female
128	+	+	+	18	+	+	-	IM	3/6/12	89	Female
131	+	+	+	18	+	+	+	IM	2/19/12	35	Male
135	+	+	+	18	+	+	+	IM	4/18/12	35	Male
139	+	+	+	18	+	+	+	GS	4/4/11	60	Male
140	+	+	+	18	+	+	+	PS	4/24/11	75	Male
156	+	+	+	18	+	+	-	IM	4/17/12	75	Female
161	+	+	+	18	+	+	+	Trauma	4/1/12	20	Female
166	+	+	+	18	+	+	+	IM	4/17/12	35	Female
169	+	+	+	18	+	+	+	Trauma	5/2/12	50	Male
176	+	+	+	18	+	+	+	IM	5/9/12	42	Female
183	+	+	+	18	+	+	-	Trauma	5/24/12	15	Male
186	+	+	+	0	-	-	-	IM	6/1/12	29	Female

IM: Internal Medicine; ICU: Intensive Care Unit; PS: Plastic Surgery; GS: General Surgery

The remaining 9.1% (2/22) of the isolates were positive for *tcdB*, *tcdA*, and *tcdC*; had no deletion of the downregulator gene *tcdC*; and were negative for the binary toxin marker genes *cdtB* and *cdtA* (Table 3).

The 2 stool samples from which these isolates were obtained tested negative for the toxigenic NAP1/027 strain: toxigenic *C*. *difficile* positive/presumptive NAP1/027 negative.

PFGE was performed on 17 isolates, and one main pattern characteristic of NAP1 strain was observed. Only two isolates showed a different pattern (isolates 106 and 186).

Analysis of the ribotyping data showed similar results with a predominant ribotype characteristic of 027 strains and only two isolates showing a different pattern (isolates 106 and 186).

## Discussion

*C*. *difficile* is an important nosocomial pathogen associated with diarrheal disease. In this paper, we present the first report of *C*. *difficile* ribotype NAP1/027 from Mexico.

One of the most used tests for the diagnosis of CDI is the ImmunoCard assay. In our study, we screened 106 patients with probable CDI, and 36 tested positive with the ImmunoCard assay. Interestingly, among the 22 patients with a positive culture for *C*. *difficile*, only 14 were positive by the ImmunoCard assay. According to the manufacturer, the ImmunoCard assay has a sensitivity of 95.2%, a specificity of 98.5%, and a negative predictive value of 99%. However, based on our results, the culture was not able to detect 12 infected patients and the ImmunoCard test missed 8 patients. Even though it was not our objective to evaluate the diagnostic tests, it seems that it is necessary to perform both for a more accurate diagnosis. Direct PCR from stool specimens is a widely used test for the diagnosis of CDI because it has high sensitivity, specificity, positive predictive value, and negative predictive value. In fact, PCR has been recommended as the preferred diagnostic test for CDI [13].

While no test is 100% sensitive and specific for the detection of *C*. *difficile*, cell cytotoxigenic and cytotoxin tests are the current gold standards. A bias of our study is that these tests are not still available in our laboratory for confirmation of the PCR results, we are already implementing this technique.

Previous work has determined that approximately one out of ten patients who acquire *C*. *difficile* will die [14] and that mortality associated with ribotype NAP1/027 infection increases with the patient’s age. A recent study has shown that 44.1% of infected patients were older than 65 years and that 8.1% of them died within 30 days after diagnosis [15]. In our study, 3 out of 22 patients (13.6%) died from CDI, with an average age of 67.6 years. If we consider all infected patients (n = 44) (36 toxin-positive patients plus 8 culture-positive, ImmunoCard-negative patients), we had a mortality rate directly attributed to CDI of 6.8% (3/44). In our study, we did not find a direct epidemiologic link between the CDI NAP1/027 cases.

*C*. *difficile* infection is also related with higher health care costs due to longer hospital stays [16]. It has been shown that hospital-acquired *C*. *difficile* infection significantly extends the duration of a patient’s stay in hospital [17]. Furthermore, *C*. *difficile* is most commonly a nosocomial infectious agent, with reservoirs being patients, healthcare workers, and the hospital environment; and the major risk for CDI is antibiotic exposure within the preceding 2–3 months [18]. The risk factors for our patients were a hospital stay of 16.1 days prior to toxin detection, average antibiotic use for 16.2 days, and a median of 3 antibiotics used.

Typing of *C*. *difficile* isolates is mostly based on one or more of three common typing schemes, including restriction enzyme analysis (the epidemic clone is type BI), PFGE (the epidemic clone is NAP1), and/or PCR ribotyping (the epidemic clone is ribotype 027). It has been described that PFGE exhibits better discriminatory power than PCR ribotyping, with *D*-values of 0.843 and 0.688, respectively [19]. In our study, we used both methodologies and both were able to show the clonal relationship of the isolates. However, PCR ribotyping is much easier to perform in the laboratory than PFGE analysis because it is less laborious.

A more severe disease presentation is more likely to be caused by ribotype NAP1/027 strains than by non-027 strains because ribotype NAP1/027 strains produce excessive amounts of toxins A and B as a result of *tcdC* deletion [4]. In our hospital, most of the isolates were ribotype NAP1/027 and were disseminated in several hospital wards; thus, special care should be taken to avoid the spread of this ribotype. In summary, the above findings represent the clonal spread of *C*. *difficile* in our institution, which mainly includes the ribotype NAP1/027 strain.

Clonal spread of *C. difficile* is a mayor concern in the healthcare environment and thus the isolation of *C. difficile* NAP1/027 requires an ongoing genetic surveillance for an early identification of new cases. Hospital policies regarding the use of clorine based disinfectants and earlier isolation precautions were reinforced after these findings.

These data confirm the emergence of *C*. *Difficile* in Mexico. Surveillance data are needed to assess the dissemination of this strain in the hospital and to establish risk factors related with the transmission of this bacterium in our healthcare facilities. This is the first report of *C*. *difficile* ribotype NAP1/027 in Mexico.
