# Correction: First Report of *Clostridium difficile* NAP1/027 in a Mexican Hospital

**DOI:** 10.1371/journal.pone.0129079

**Published:** 2015-05-28

**Authors:** Adrián Camacho-Ortiz, Daniel López-Barrera, Raúl Hernández-García, Alejandra M. Galván-De los Santos, Samantha M. Flores-Treviño, Jorge M. Llaca-Díaz, Héctor J. Maldonado-Garza, Francisco J. Bosques-Padilla, Elvira Garza-González

The seventh author’s name is spelled incorrectly. The correct name is: Héctor J. Maldonado-Garza.
